# Identification of three immune molecular subtypes associated with immune profiles, immune checkpoints, and clinical outcome in multiple myeloma

**DOI:** 10.1002/cam4.4221

**Published:** 2021-08-21

**Authors:** Guangtao Gao, Mengkun Fang, Peipei Xu, Bing Chen

**Affiliations:** ^1^ Department of Hematology The Affiliated Drum Tower Hospital of Nanjing University Medical School Nanjing Jiangsu China

**Keywords:** immune checkpoint, immune metagenes, immune molecular subtypes, multiple myeloma, survival prognosis

## Abstract

**Purpose:**

To identify the immune molecular subtype for MM to help achieve individualized and precise targeted therapy.

**Methods:**

The GDC API was used to download the TCGA‐MM profile dataset, which contains 859 samples in total, all of which were anterior to the standard treatment after diagnosis. Moreover, 282, 298, and 258 samples were stage I, stage II, and stage III separately. We used the immune gene expression profile for consistent clustering; and used the R software package ConsensusClusterPlus to sort the immune molecular subtypes. Correlation between subtypes and clinical features, immunity, and prognosis was then analyzed.

**Results:**

A total of 859 tumor samples were separated into these three subtypes, which were not meaningfully related to age or sex but showed a remarkable association with stage. The results suggested that obvious differences in immune metagene expression and expression of 10 immune checkpoint genes appeared among the three subtypes.

**Conclusion:**

The three subtypes are distinctly different in terms of immune metagenes, immune checkpoint molecules, and clinical prognosis. The discovery of the immune microenvironment of MM could further reveal the strategy for immunotherapy in MM and provide a promising candidate prognostic tool for survival.

## Graphical abstract

Multiple myeloma (MM), being the second most common hematological malignancy, remains incurable for a large part of patients, hence precision medicine and individualized treatment are urgently required to improve the clinical conditions. Faced with the complexityof treatment options for multiple myeloma, histopathological standards cannot provide plenary information. The immunomolecular standards could improve clinical outcomes with current treatments and speed up the therapeutic development through more accurate patient classification. The Cancer Genome Atlas (TCGA) cohort was utilized to investigate the immune function of the tumor microenvironment and its clinical correlation with multiple myeloma. The immune‐related research and clinical features showed that the three subtypes were all significantly associated with clinical stage. In contrast with other subtypes, C2 subtype had the worst survival prognosis. These findings of immunological microenvironment in tumors have the potential to ameliorate the management of patients with MM in several ways.

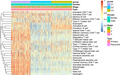

## 1 INTRODUCTION

Multiple myeloma (MM) is the second most common hematological malignancy after non‐Hodgkin lymphoma. It is characterized by the proliferation of clonal plasma cells in the bone marrow, it remains an incurable disease.^1^, ^2^ More than 100,000 patients worldwide are diagnosed with MM each year which accounts for approximately 10% of all hematological malignancies.^3^, ^4^ The increasing incidence and prevalence of MM make it a significant and growing healthcare challenge with a relapsing course.

The diagnosis and treatment of MM has improved significantly, and there are many available treatments including alkylating agents, deacetylase inhibitors, immunomodulatory agents, proteasome inhibitors, and monoclonal antibodies. MM armamentarium is widely used and almost every patient can acquire adequate strategies and optimal sequences of drugs.^3^, ^4^, ^5^, ^6^ Given our increasing understanding of the diagnosis and treatment of MM, now would seem an appropriate time to provide a more robust classification system for MM, which will pave the way for personalized medicine and improve the quality of life and survival of patients with MM.^7^

Immunotherapy is considered the fifth pillar of cancer care and represents a paradigm shift in oncology treatment.^8^, ^9^ As a result of advanced molecular diagnostic platforms and key discoveries on immune mechanisms, immunotherapy has revolutionized the field of cancer therapeutics and generated considerable excitement for the treatment of almost all types of cancers.^10^, ^11^, ^12^ However, the management of MM in clinical trials remains challenging despite the enormous advances in immunotherapy and patients with MM treated with immunotherapy have exhibited diverse remission rates within cohorts.^13^ The reasons for individual differences in cancer immunotherapy have been attributed to several factors, including differing antigen specificity and expression levels, immune competency, and diversity.^7^, ^14^, ^15^ The intricate tumor immune microenvironment plays an important role in the effectiveness of immunotherapy; however, the relationship between the tumor immune microenvironment in MM and clinical prognosis is currently unclear. Hence, it is essential to fully explore the immune status of patients, to confirm the molecular subtypes of MM, and to further improve treatment outcomes in patients with MM.

The purpose of this study was to investigate the overall immune status of patients with MM and its clinical significance. By screening the immune gene expression data from the TCGA database, we identified three molecular subtypes of MM.^7^, ^16^, ^17^ We then compared clinical features, immune landscape, and finally, our analysis results were validated using external datasets. These findings are of great significance for the individualized treatment of MM and may guide the treatment principles in future clinical trials.^18^

## 2 MATERIALS AND METHODS

### 2.1 Data collection and processing

TCGA‐MM profile dataset was downloaded from TCGA and contained a total of 859 samples, all of which were samples anterior to the standard treatment after diagnosis. Moreover, 282, 298, and 258 samples were stage I, stage II, and stage III separately. Detailed clinical information, including age, sex, tumor type, and tumor stage was also collected from the study, as listed in Table 1. We then matched the expression profile with the clinical follow‐up samples and chose these as the sample set for the study. Furthermore, we extracted the expression profiles of immune gene sets and selected the expression levels in each sample that were greater than 0. Eventually, 5000 genes met the inclusion criteria which means sample with more than 30% of the genes was included as an immune gene for this study.^19^

**TABLE 1:** Relationship between three subtypes and clinical characteristics (χ^2^ test)

	C1	C2	C3	*p*. overall
	*N* = 272	*N* = 295	*N* = 292	
Age	64.0 (11.2)	62.8 (10.2)	62.1 (10.4)	0.098
Gender:				0.406
Female	105 (38.6%)	122 (41.4%)	129 (44.2%)	
Male	167 (61.4%)	173 (58.6%)	163 (55.8%)	
Type:				0.003
Primary	235 (86.4%)	264 (89.5%)	277 (94.9%)	
Recurrent	37 (13.6%)	31 (10.5%)	15 (5.14%)	
Stage:				<0.001
I	83 (31.3%)	82 (28.5%)	117 (41.1%)	
II	98 (37.0%)	86 (29.9%)	114 (40.0%)	
III	84 (31.7%)	120 (41.7%)	54 (18.9%)	

The GSE136400 dataset of the GPL570 platform which contained 1293 standard samples with survival information as listed in Table 2 was downloaded using the R package GEOquery.

**TABLE 2:** Summary descriptive of the GEO database

	C1	C2	C3	*p*. overall
	*N* = 800	*N* = 133	*N* = 360	
OS	0.39 (0.49)	0.53 (0.50)	0.41 (0.49)	0.009
PFI	0.49 (0.50)	0.61 (0.49)	0.53 (0.50)	0.031
Gender:				0.397
Female	297 (37.1%)	56 (42.1%)	145 (40.3%)	
Male	503 (62.9%)	77 (57.9%)	215 (59.7%)	
Stage:				0.004
I	355 (44.5%)	38 (28.6%)	143 (40.4%)	
II	254 (31.8%)	47 (35.3%)	109 (30.8%)	
III	189 (23.7%)	48 (36.1%)	102 (28.8%)	
OS time	2860 (1335)	2229 (1423)	2648 (1316)	<0.001
PFI time	1603 (929)	1223 (875)	1505 (941)	<0.001

Eventually, we appraised and quantified the immune and matrix scores for each sample using the R package.

### 2.2 Molecular subtypes screening according to immune genes

We screened the molecular subtypes using the immune gene expression profile for consistent clustering and the R software package ConsensusClusterPlus. The Euclidean distance was used to calculate the similarity distance between samples, while K‐means was used for clustering. We then sampled 80% of the samples using a resampling scheme that was executed 100 times. Using the cumulative distribution function (CDF), we figured out the optimal number of clusters. In the end, the R package sigclust was utilized to further analyze the clustering significance between these subtypes.

### 2.3 The correlation between subtypes and clinical features

The development of the disease is closely linked to different clinical features. By analyzing the correlation between subtypes and clinical features, we could further understand the correlation between subtypes and disease development. Then the correlation between the subtypes and age, grade, and stage was observed in the light of the clinical follow‐up data of the patients.

### 2.4 The correlation between subtypes and immunity

To investigate the correlation between the immune metagenes and subtypes, we selected 13 types of immune metagenes that are involved in the immune process. Based on the relationship between the immune components of tumor tissue and prognosis, we further studied the correlation between the matrix, immune landscape, and molecular subtypes. We further evaluated the differences in the scores of the subtypes by utilizing variance analysis.

### 2.5 The correlation between subtypes and prognosis

We utilized K–M to evaluate the prognostic differences between the different subtypes after we processed the follow‐up data from the sample follow‐up information.

### 2.6 Other statistical methods

To study the relationship between the molecular subtypes and conventional clinical variables, chi‐square test and exact test of Fisher's were utilized. Besides, the log‐rank test and Kaplan–Meier curves were utilized when we compared the OS rates of all molecular subtypes. Meantime, all the statistical tests were two‐sided tests and we utilized R software for statistical analysis.

## 3 RESULTS

### 3.1 Identification of three immune molecular subtypes of MM based on immune profiles

**FIGURE 1:**
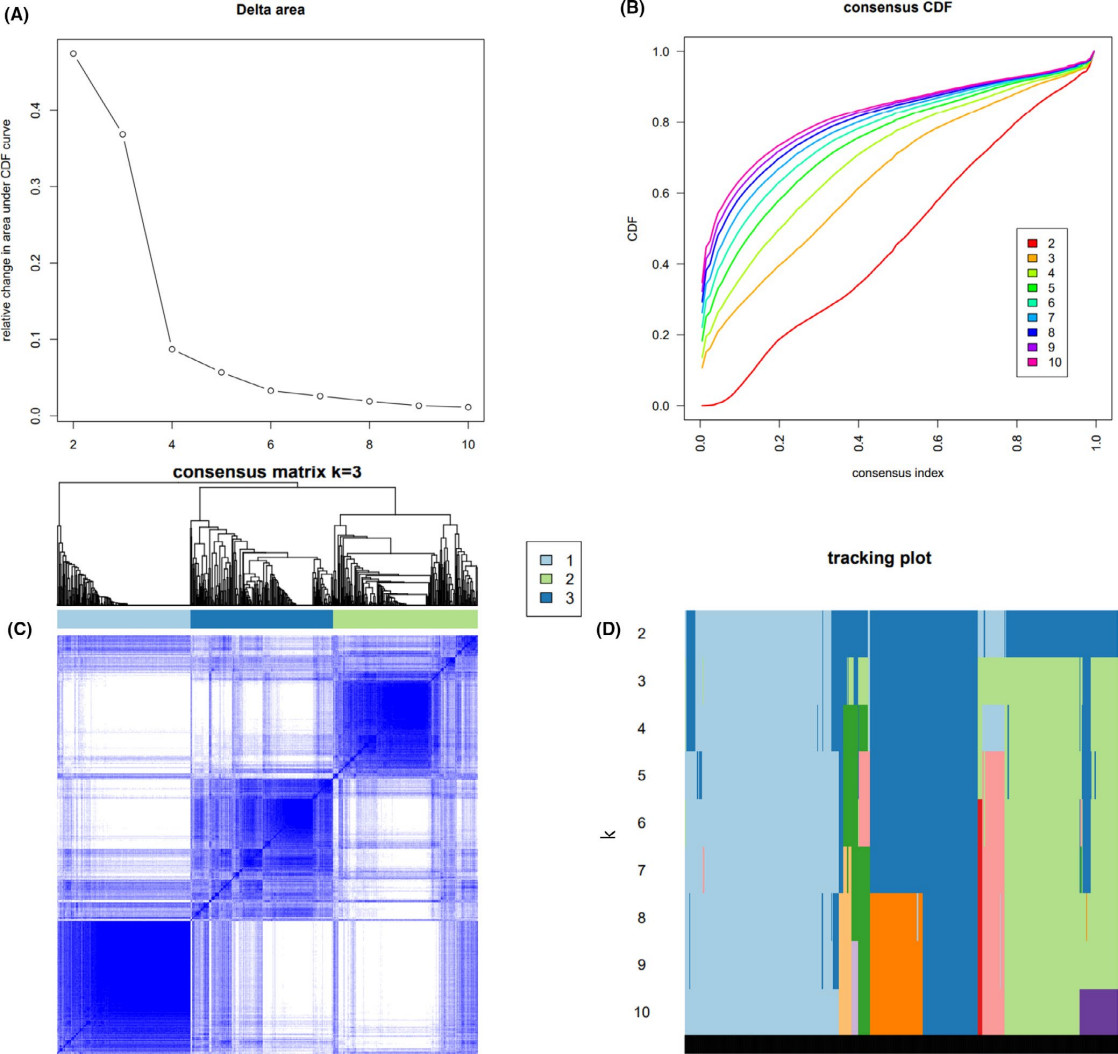
Identification of MM subtypes based on immune genes. (A) CDF curve; different colors reflect different cluster numbers, the horizontal axis represents the consensus index, the vertical axis stands for cumulative distribution function (CDF), and a bigger AUC indicates better clustering. (B) CDF delta area curve of consensus clustering, indicating the relative change in area under the cumulative distribution function (CDF) curve for each category number k compared with *k* − 1. The horizontal axis represents the category number *k*, and the vertical axis represents the relative change in area under CDF curve. (C) Heatmap of sample clustering at consensus *k* = 3; (D) Heatmap of sample clustering at consensus *k* = 4

To identify the MM immune molecular subtypes in the TCGA cohort, the gene expression profiles of 782 immune‐related genes were considered.^20^ Using ConsensusClusterPlus, the most favorable number of clustering was achieved when *k* = 3 (Figure 1) according to the cumulative distribution function curves of the consensus score.^20^, ^21^

### 3.2 Correlation between the three molecular subtypes and clinical characteristics

As shown in Table 1, we analyzed the correlation between the three subtypes, age, sex, and tumor type and tumor stage. Three subtypes had almost no relationship with age or sex, but showed a notable correlation with tumor type and stage. For instance, the recurrence of C1 subtype is notably greater than other subtypes; meanwhile, there were more stage Ⅲ samples of C2 subtype than those of the other subtypes.

### 3.3 Correlation between the three subtypes and immunity

**FIGURE 2:**
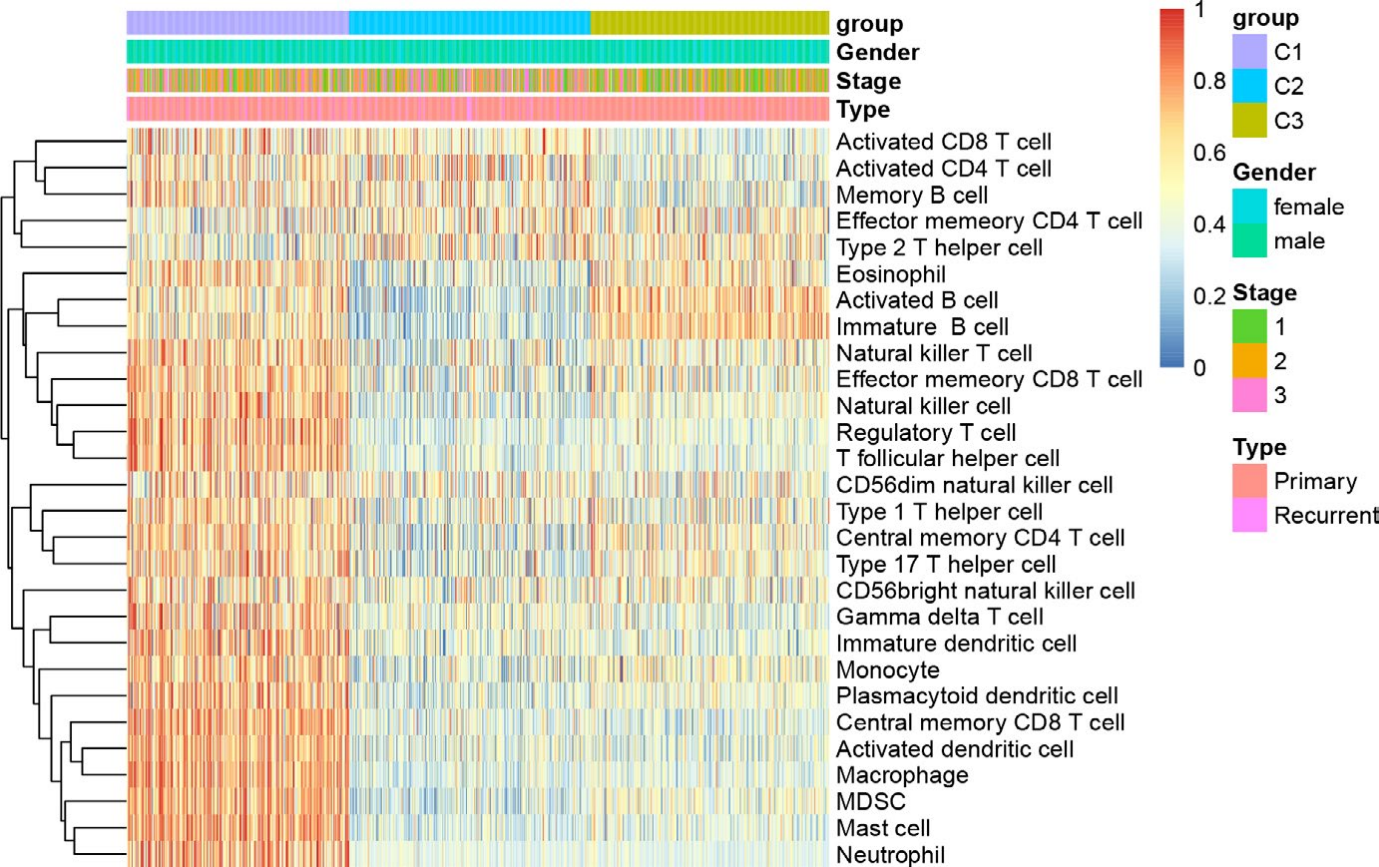
Immune profiles of the three molecular subtypes in the TCGA‐MM cohort. Gene expression score of 28 groups of immune metagenes in 4 molecular subtypes of ovarian cancer. In the heat map of gene expression, red represents high expression and blue represents low expression

To analyze the relevance between the three subtypes and immune microenvironment, we analyzed the relevance between the three subtypes and 28 immune metagenes.^22^ The results revealed that the expression of the 28 immune metagenes is quite different; the majority of which were highly expressed in the C1 subtype, whereas almost all the 28 immune metagenes exhibited low expression in the C3 subtype (Figures 2, S1‐S3).

### 3.4 Relationship between the three subtypes and the expression of 10 immune checkpoint genes

**FIGURE 3:**
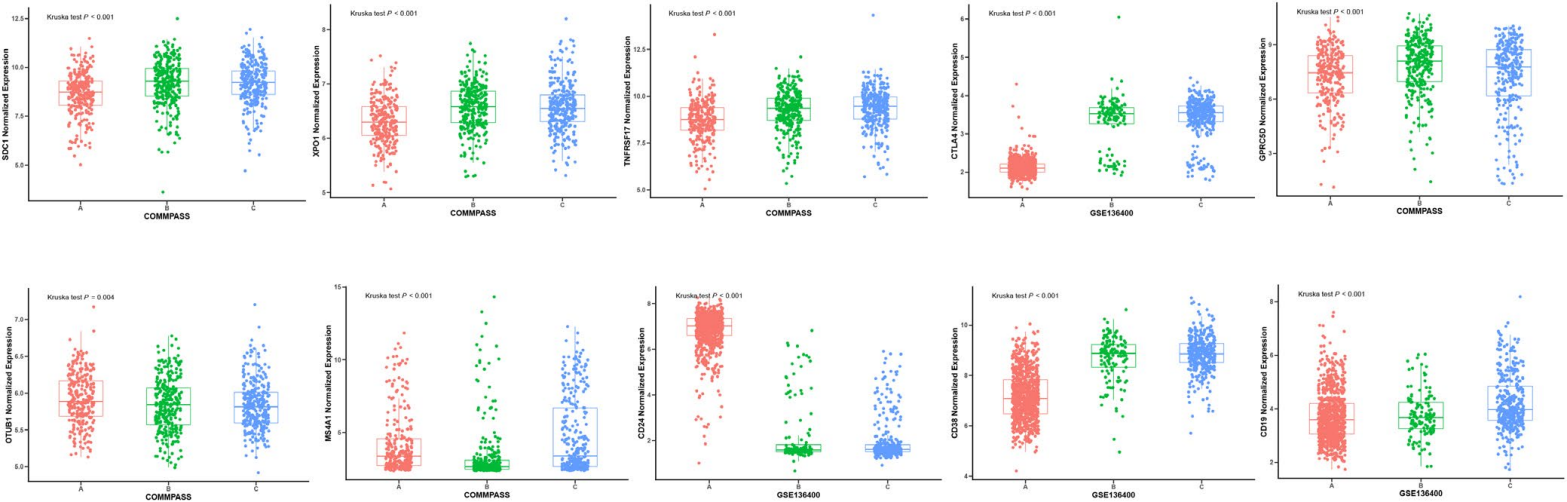
Expression distribution of 10 immune checkpoint genes in 3 subtypes in the TCGA‐MM cohort

We further studied the relationship between the expression of 10 immune checkpoint genes and the three subtypes. The expression levels of *SDC1*, *XPO1*, *TNFRSF17*, *GPRC5D*, and *CD38* in C1 subtype were significantly lower than those in other subtypes, while the C3 subtype showed higher expression levels of *TNFRSF17* and *CD38* (Figure 3). Statistical significance was set at *p* < 0.05.

### 3.5 Prognosis differences between the three subtypes

**FIGURE 4:**
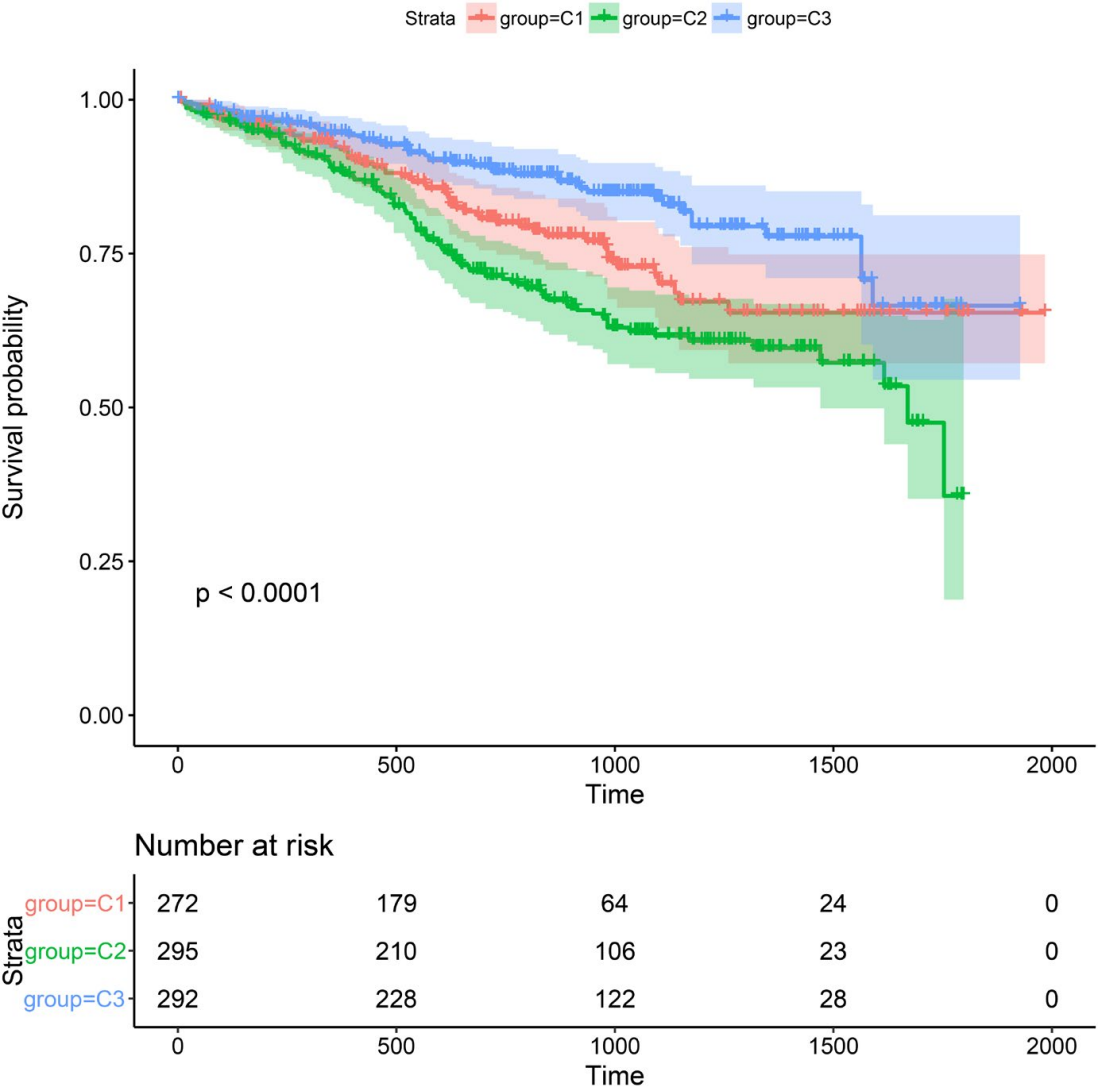
Survival analysis of the three MM subtypes. KM curves showing prognostic relationship of 3 subtypes; The *p*‐value was calculated using the log‐rank test, by comparing the overall survival of 3 subtypes. The abscissa represents survival time (d) and the ordinate represents survival probabilities

The Kaplan–Meier method was used to study the prognostic differences between the three subtypes and further explore the correlation between the three subtypes and prognosis.^23^ There was an obvious contrast between the three subtypes regarding prognosis: the C2 subtype had the worst prognosis and the C3 subtype showed better prognosis than the other subtypes (Figure 4).

### 3.6 Validation of external datasets

**FIGURE 5:**
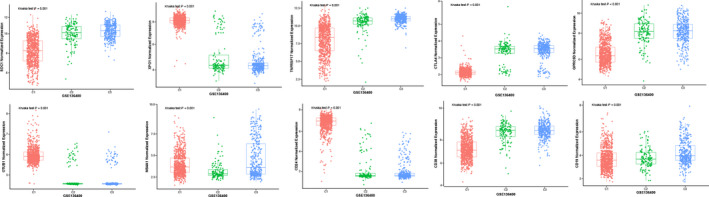
Validation of external datasets. Expression distribution of 10 immune checkpoint genes in 3 subtypes in the validation set

**FIGURE 6:**
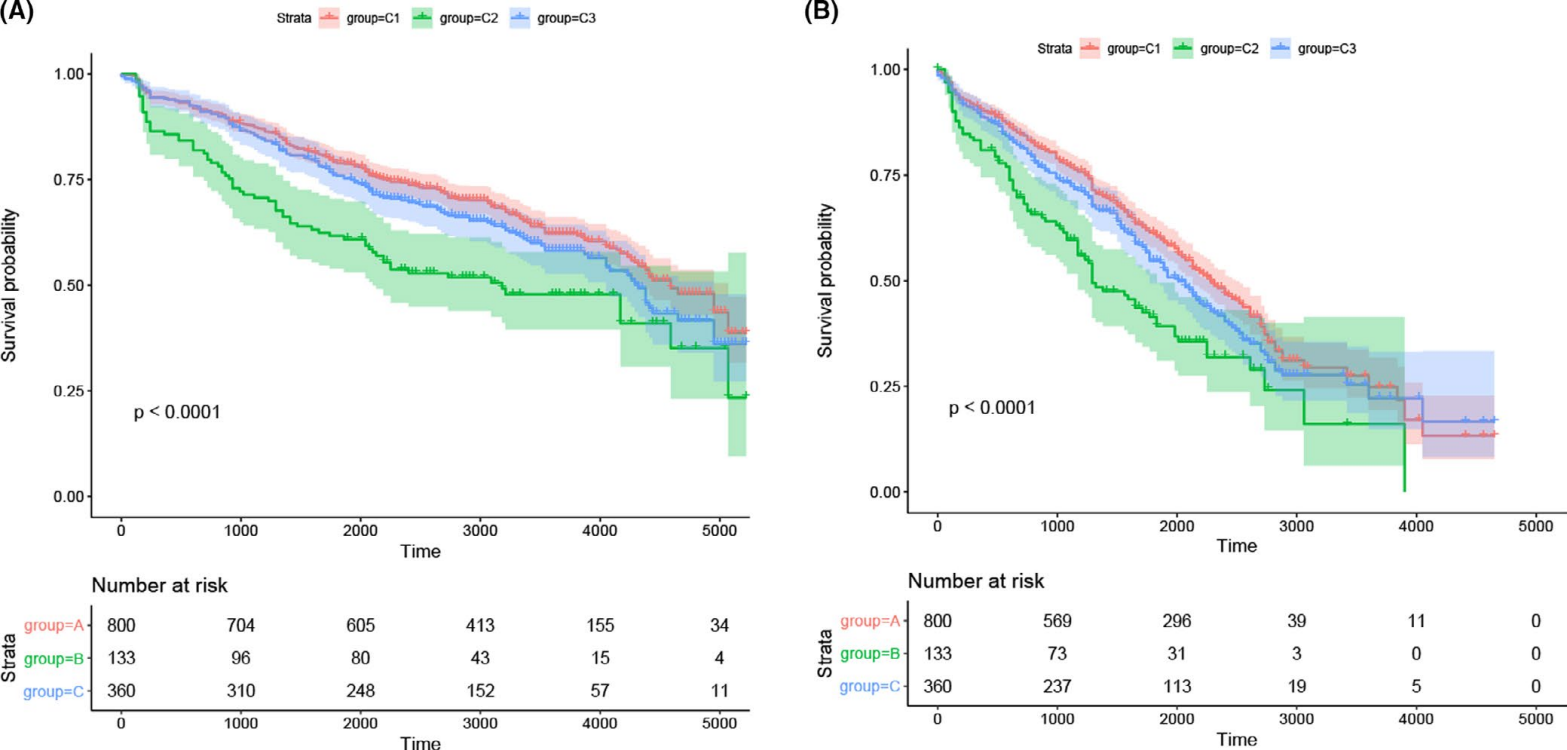
Validation of external datasets. (A) Overall survival analysis of the three MM subtypes. (B) Progression free survival analysis of the three MM subtypes

Within the HSIC Lasso framework, we performed feature selection and selected 120 genes.^24^ To further identify the three subtypes, GSE136400 standard data were downloaded from the GEO database, which included 1293 samples. As shown in Figure 5, 7 of 10 genes demonstrated a similar expression to the 10 immune checkpoint genes. Based on the analysis of prognostic differences (Figure 6), the results of the validation dataset were consistent with those of the TCGA cohort.

## 4 DISCUSSION

With the emerging immunotherapy for MM,^5^, ^8^, ^25^, ^26^ histopathological criteria cannot adequately provide treatment recommendations; therefore, an immune molecular taxonomy has the potential to improve outcomes and accelerate therapeutic development.^16^, ^27^, ^28^ Increasing research has focused on exploring the molecular subtypes of various types of tumors based on genome‐wide profiles or multi‐omics.^16^, ^19^, ^29^, ^30^, ^31^, ^32^ These strategies will lead to better treatment options, focused on the underlying biology of each specific subtype. These findings provide new insights into the diagnosis and treatment of MM.

Despite comprehensive survival amelioration and the availability of new drugs in the past two decades, MM is still an incurable disease.^33^ However, treatment for MM continues to develop as a result of many emerging immunotherapies that may achieve treatment breakthroughs.^25^, ^34^ The first immunotherapy for MM was an allogeneic stem cell transplant, which remains a routine treatment for the long‐term management of high‐risk diseases.^35^ In the mid‐2000s, immunomodulatory drugs designed to improve the immunomodulatory and anticancer properties and tolerability profiles of treatments; such as thalidomide, were shown to be effective in MM and substantially improved survival.^36^ The next generation of immunotherapies for MM comprises monoclonal antibodies,^37^ chimeric antigen receptor T cells,^38^ bispecific antibodies,^39^ antibody drug conjugates,^40^ and checkpoint inhibitors.^41^ Although several other novel receptors have been identified, B‐cell maturation antigen (BCMA) is still the predominant target for emerging treatments.^42^

Nevertheless, MM appears to be able to escape immunotherapy as seen with chemotherapy, due to the intimate relationship with the cells in the bone marrow microenvironment, which supports multiple aspects of the tumor.^25^, ^43^ Thus, not all therapies can successfully treat MM, and some may even be harmful. Therefore, it is of great clinical significance to screen for immune molecular subtypes of MM, which will contribute to the individualization of immunotherapy.

The superiority of the research is predominantly to investigate the global immune spectrum, which can contribute to more features about the immune landscape in MM. Finally, we identified three gene expression subtypes according to global immune genes in the TCGA‐MM cohort and confirmed them in the external dataset GSE136400.

The overall immune profiles were significantly different among the three molecular subtypes, with different high‐expressing immune‐related cells. These findings suggested that the C1 subtype was linked to an enriched immune status in the tumor microenvironment, which can be described as EIME (enriched immune status in the tumor microenvironment).^20^ Meanwhile, the C3 subtype showed a reduced immune status, and the C2 subtype was the intermediate type. Importantly, we concluded that the immune phenotype has a greater influence on survival which may allow a more accurate classification of patients and contribute to the realization of personalized medicine. Regarding survival probability, the prognosis of the C2 subtype was the poorest while the C1 subtype showed the best prognosis. This suggested that the immune‐enhanced subtypes may correspond to the best prognosis in MM. In addition, we could draw the conclusion that an abundance of infiltrating lymphocytes correlates with favorable prognosis and the activation of antitumor adaptive immune responses can inhibit tumor development.^44^

As a fresh hallmark of immunotherapy for MM, immune checkpoint blockade therapy has shown unexpected antitumor effects in patients with relapsed and/or refractory MM, which motivated us to find more potential immune checkpoints.^45^, ^46^ Hence, we further explored the correlation between the three subtypes and 10 immune checkpoint genes (*MS4A1*, *GPRC5D*, *OTUB1*, *XPO1*, *SDC1*, *CD19*, *CD38*, *CTLA4*, *CD24*, *and TNFRSF17*), which mainly encode high‐interest therapeutic targets. Currently, daratumumab, a monoclonal antibody developed for *CD38*, has been approved for the treatment of MM.^47^ Our results suggested that the expression levels of *SDC1*, *XPO1*, *TNFRSF17*, *GPRC5D*, and *CD38* in the samples of the C1 subtype were significantly lower than those in other subtypes, while the C3 subtype showed higher expression levels of *TNFRSF17* and *CD38*. In summary, these observations may help physicians choose precise immune checkpoint blockade and treat patients with personalized medicine.

## 5 CONCLUSION

In conclusion, three immune subtypes of MM were identified using global immune gene expression profiles by exploring TCGA databases. The three subtypes were distinctly different in terms of immune metagenes, immune checkpoint molecules, and clinical prognosis. The discovery of the immune microenvironment of MM could further inform the strategy of immunotherapy in MM and provide a promising candidate prognostic tool for survival.

## ETHICS APPROVAL AND CONSENT TO PARTICIPATE

The patient data in this work were acquired from the publicly available datasets whose informed consent of patients was complete.

## CONFLICT OF INTEREST

The authors declare that they have no conflict of interest.

## Supporting information

Supplementary Material

## Data Availability

Not applicable.
