# Supplementary material for: Identification of three immune molecular subtypes associated with immune profiles, immune checkpoints, and clinical outcome in multiple myeloma
Source: Cancer Med. 2021 Aug 21;10(20):7395–403. doi: 10.1002/cam4.4221 (PMC8525096; doi:10.1002/cam4.4221)
Supplement: Supplementary file 1 — Supplementary Material [file CAM4-10-7395-s001.docx]

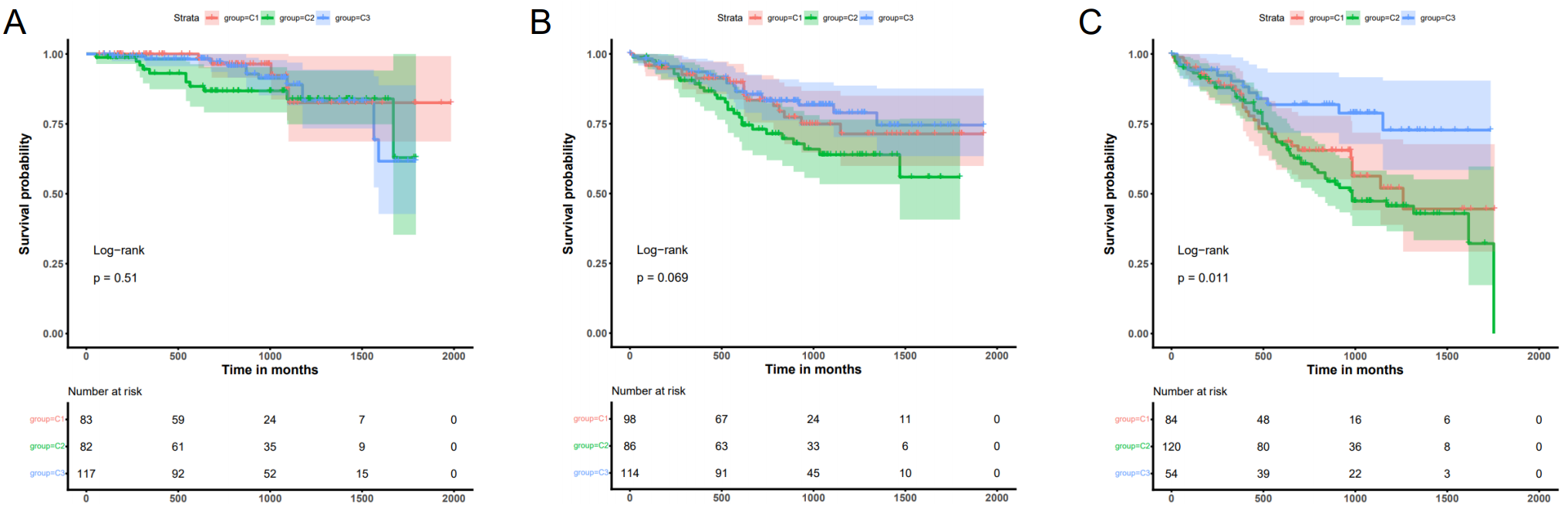


**Figure S1** Survival analysis of the three MM subtypes regarding the impact of imbalance of clinical stage. Figure A, B, C represent the survival analysis of the three MM subtypes in stage Ⅰ, Ⅱ, Ⅲ. KM curves showing prognostic relationship of 3 subtypes; The P-value was calculated using the log-rank test, by comparing the overall survival of 3 subtypes. The abscissa represents survival time. (d) and the ordinate represents survival probabilities.


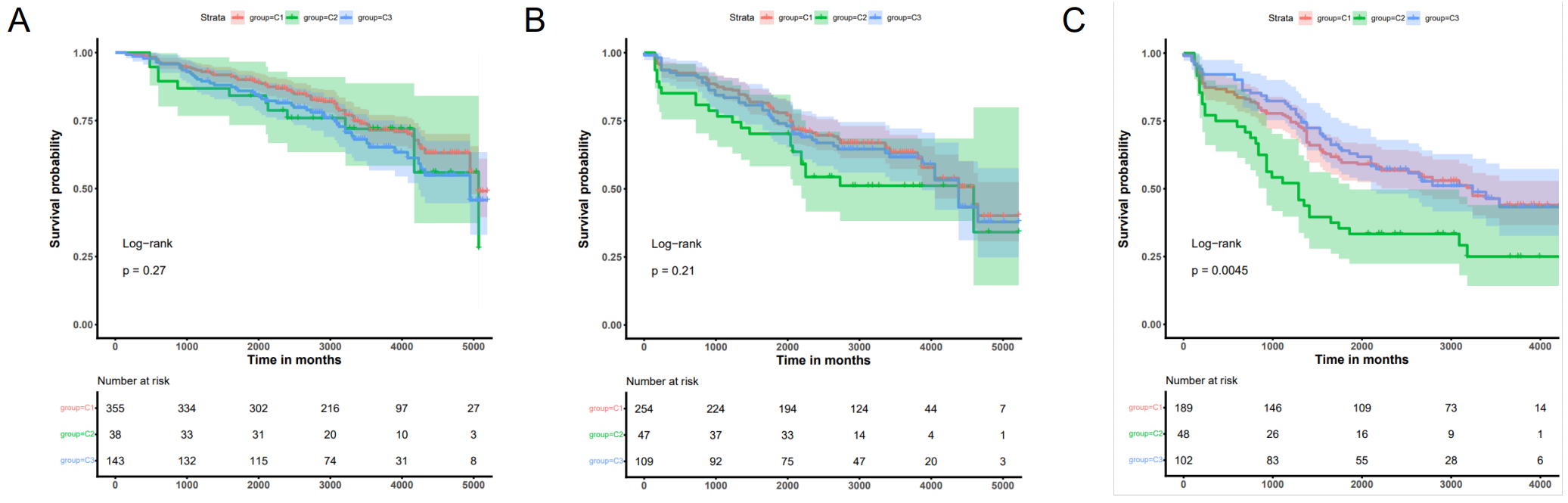


**Figure S2** Validation of external datasets. Figure A, B, C represent the overall survival analysis of the three MM subtypes in stage Ⅰ, Ⅱ, Ⅲ.


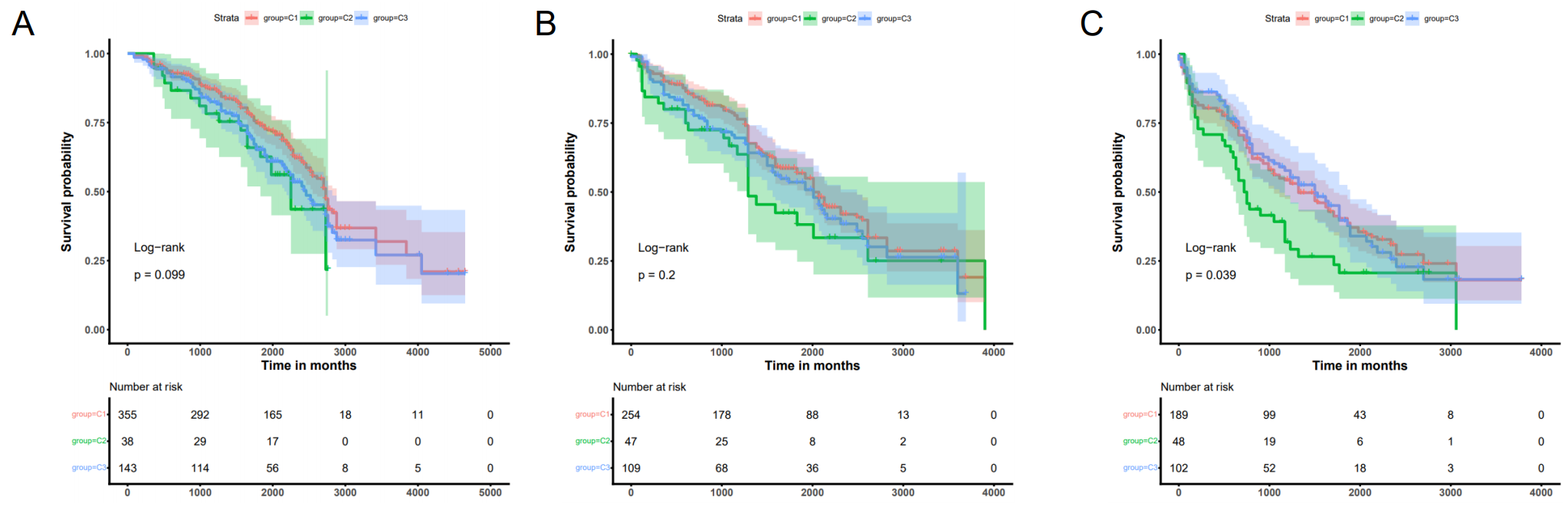


**Figure S3** Validation of external datasets. Figure A, B, C represent the progression free survival analysis of the three MM subtypes in stage Ⅰ, Ⅱ, Ⅲ.
